# A Magnetic-Driven Multi-motion Robot with Position/Orientation Sensing Capability

**DOI:** 10.34133/research.0177

**Published:** 2023-06-21

**Authors:** Liwen Zhang, Song Zhao, Xinzhao Zhou, Xueshan Jing, Yu Zhou, Yan Wang, Yantong Zhu, Xiaolin Liu, Zehui Zhao, Deyuan Zhang, Lin Feng, Huawei Chen

**Affiliations:** ^1^ School of Mechanical Engineering and Automation, Beihang University, Beijing, China.; ^2^ Beijing Advanced Innovation Center for Biomedical Engineering, Beihang University, Beijing, China.

## Abstract

Miniature magnetic-driven robots with multimode motions and high-precision pose sensing capacity (position and orientation) are greatly demanded in in situ manipulation in narrow opaque enclosed spaces. Various magnetic robots have been carried out, whereas their deformations normally remain in single mode, and the lack of the robot’s real-time status leads to its beyond-sight remagnetization and manipulation being impossible. The function integration of pose sensing and multimode motion is still of challenge. Here, a multimotion thin-film robot is created in a novel multilayer structure with a magnetic-driven layer covered by a heating-sensing conductive layer. Such a heating-sensing layer not only can segmentally and on-demand heat the magnetic-driven layer for in situ magnetization reprogramming and multimode motions but also can precisely detect the robot’s pose (position and orientation) from its electrical-resistance effect by creating a small deformation under preset magnetic fields. Under the integration of reprogramming and sensing, necessary multimode motions, i.e., swimming, rolling, crawling, and obstacle-crossing, are achieved under a reprogramming field *B_Repr_* of 10 mT, and high-precision poses sensing with an accuracy of ± 3 mm in position and ± 2.5° in orientation is obtained even under a low magnetic strength of *B_Sens_* of 5 mT, which combined help realize accurate out-of-sight manipulations in the enclosed space environment. Finally, a gastroscope robot for stomach drug delivery has been demonstrated for more gastrointestinal medical treatments.

## Introduction

Magnetic-driven robots with sophisticated and accurate manipulations in confined and enclosed spaces have attracted growing attention for their potential applications in medical treatments of gastroscopy and minimally invasive surgery [1–6]. Although various magnetic robots have been carried out, their deformations under external magnetic field commonly remain in single mode of rolling or crawling due to the irreversible premagnetization, which makes the robot unable to reshape into corresponding deformations to adapt to complex environments [7–16]. Recently, smart materials in soft magnetic composites with 3-dimensional 3D programmable magnetization have been developed to realize multimode motions for diverse biomimetic motorial capabilities [17–26].

The composite film is made from an elastomer and magnetic particles encapsulated by a phase change polymer, which can be temporarily melted by transient laser heating and the orientation of the magnetic particles can be realigned upon the change of programming magnetic field. However, once these magnetic robots enter an opaque and closed environment, the external laser can hardly be accurately applied to the robot, which may fail the magnetic reprogramming and multimode motions to adapt to the complex environment [27,28].

The absence of the magnetic robot’s status in position and orientation greatly limits its medical applications in human body under an out-of-sight environment and makes the in situ reprogramming and accurate movements infeasible. Several methods have been utilized to achieve the pose sensing of robot with mapped coil sensors and radio frequency identification, where the position accuracy still needs to be improved and the orientation of robots are still unachievable [29–31]. Magnetic particle inspection with advantages of high space accuracy and rapid detecting has been introduced for the sensing of magnetic robots [32,33], whereas its requirements of ultrahigh magnetic strength in large space and sophisticated controlling and detecting systems make the robot pose sensing high cost. It is still a challenge to integrate multimodal motion and pose sensing capabilities on a single robot, which could further expand its applications in medical treatment for human [34–41].

Here, a sensing-actuating integrated reprogrammable magnetic robot has been promoted, where a layered structure has been designed with a magnetic-driven layer covered by a heating-sensing conductive layer and fabricated with 4-dimensional printing methods. The heating-sensing layer can segmentally and on-demand heat the magnetic-driven layer for in situ magnetization reprogramming to realize multimode motions. Its electrical-resistance effect can also detect the robot’s pose status, which could in turn help the magnetic reprogramming perform in an out-of-sight environment. The controlling and sensing methods for reprogramming and pose detecting have been presented. This reprogrammable magnetic robot with pose sensing and multimode motions capacities could provide more complex functions for gastrointestinal application and paves the way for the further development of micro magnetic soft robots.

## Results

### The magnetic robot with complex and accurate out-of-sight manipulations for gastroscopy

**Fig. 1.**
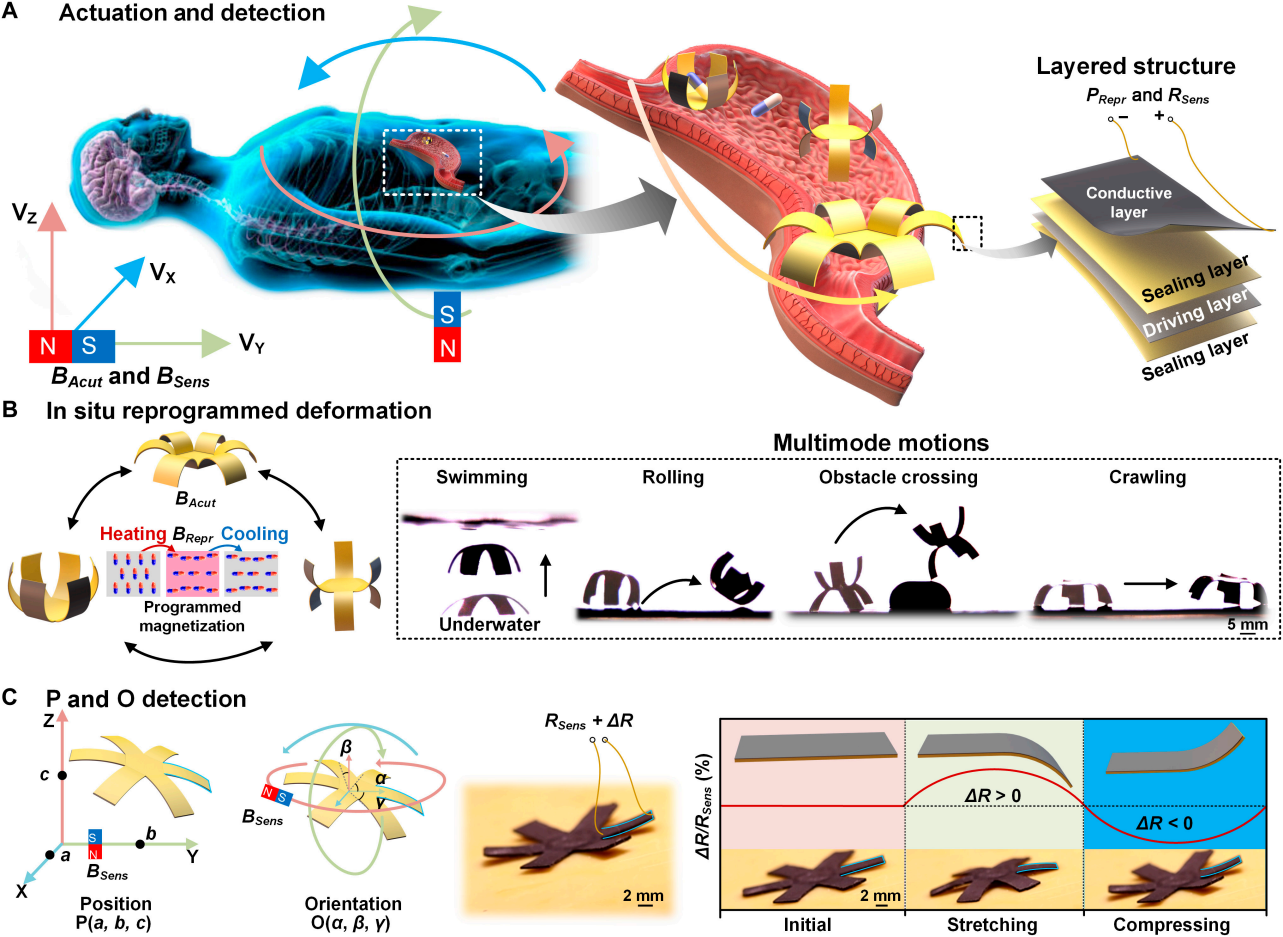
Magnetic actuated robot with in situ reprogrammable multimode movements and pose sensing capacity for gastroscopy. (A) The conceptual design of the gastroscopic robot with the capacity of multimode motions and pose sensing to meet the requirement of complex and out-of-sight manipulations in the narrow digestive system. The robot’s actuated movements, in situ reprogramming, and pose sensing are realized by an external moveable magnetic field. Its layered structure is constructed with a conductive layer and a reprogrammable magnetic driving layer between 2 sealing layers. (B) The multimode motions of the robot are realized by in situ reprogrammed deformations of the robot. Its magnetic driving layer is sectional and can be selectively heated by external electricity to turn into liquid state. A reprogramming magnetic field *B_Repr_* is exerted on the liquid magnetic driving layer to achieve remagnetization. Then, various robot deformations and motions are achieved under actuating magnetic field *B_Actu_*. (C) The out-of-sight pose sensing (position and orientation) of the robot is realized by measuring the change of electric resistance *R_Sens_* of the conductive layer under different external sensing magnetic field *B_Sens_*. With linearly and circularly moving *B_Sens_*, the change of *R_Sens_*, i.e., *ΔR*, induced by the robot’s deformation is measured to denote the robot pose with the position of P(*a*, *b*, *c*) and orientation of O(*α*, *β*, *γ*).

Gastroscopy in the narrow digestive system requires accurate and complex manipulations to deliver drugs or accomplish biopsy, where an agile millirobot could be suitable for such medical applications for improved medical experience with less pain and injury. To meet these requirements, a magnetic-driven robot is designed to perform various multimode motions for different medical operations under the control of the external actuating magnetic field *B_Actu_* (Fig. 1A, left). To realize out-of-sight medical operations in the digestive system, a pose-sensing function is also necessary for the robot, including the perception of position and orientation (Fig. 1A, middle). All these demands need the robot to be capable of in situ reprogramming, multimode moving, and pose sensing. Thus, an octopus-inspired 6-claws robot is carried out with a layered film of a carbon-based conductive layer and a reprogrammable magnetic driving layer (Fig. 1A, right). The magnetic driving layer is made with heat-induced phase-changeable material between liquid and solid, where the robots’ each arm can be segmentally and selectively heated into liquid state by the conductive layer with electricity power *P_Repr_*. This liquid-state magnetic driving layer is then remagnetized into different magnetic directions under external reprogramming magnetic field *B_Repr_*, and varied modes of motions can be achieved such as swimming, rolling, obstacle crossing, and crawling (Fig. 1B). Moreover, to realize the robot’s in situ reprogramming and multimode movements, pose sensing becomes indispensable due to the out-of-sight feature of the digestive system. By specially choosing the conductive layer material with strain-resistance effect, a varied electric resistance *ΔR* could form under small deformation with the external sensing magnetic field *B_Sens_*, which could denote the robot’s position P(*a*, *b*, *c*) and orientation O(*α*, *β*, *γ*) (Fig. 1C). Such magnetic driven robot provides more complex and precise manipulations for gastroscopy.

### Magnetic driven material fabrication and in situ reprogramming control

**Fig. 2.**
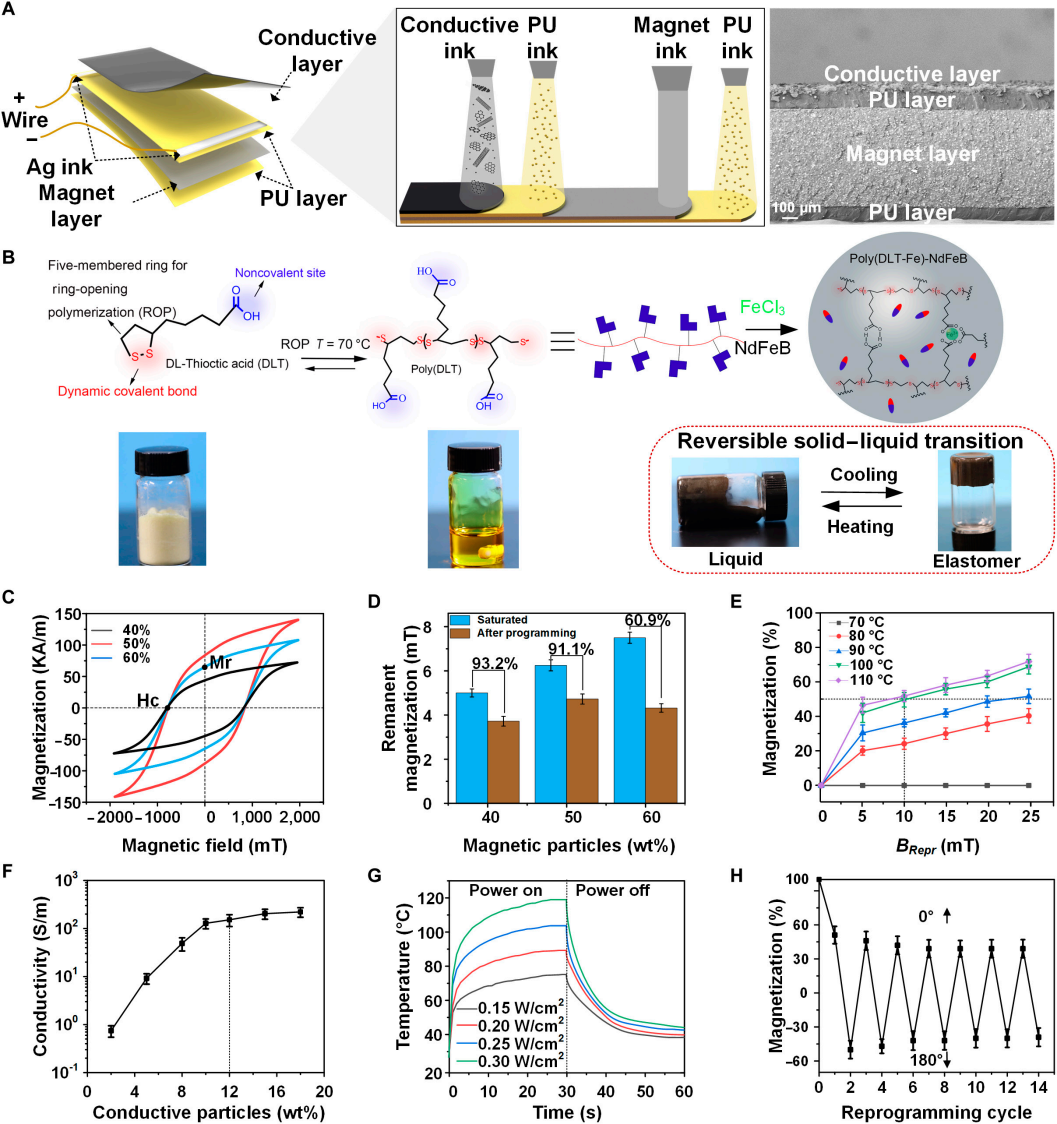
Design of the in situ reprogrammable magnetic drive and material properties optimization. (A) Layered structure for in situ reprogrammable magnetic drive with a carbon-based (carbon nanotube and graphite) conductive layer and a solid–liquid reversible magnetic layer sealed by 2 PU layers. Two Ag electrodes align below the conductive layer. 3D printing and aerojet printing methods have been utilized for layered film fabrication with conductive ink, PU ink, magnetic ink, and Ag ink. Cross-sectional SEM images of the layered film. (B) The chemical synthesis route of the heat-induced solid–liquid reversible magnetic ink. DLT is treated with thermal-initiated ring-opening polymerization at 70 °C to form poly(DLT) and then introduced with Fe^3+^ to turn into elastomer, which could be reversed between solid–liquid by heating and cooling. NdFeB magnetic microparticles are uniformly dispersed into the polymer to form poly(DLT-Fe)-NdFeB to be magnetic actuatable. (C) Magnetic hysteresis curves of the magnetic ink with NdFeB weight fractions of 40%, 50%, and 60%. (D) The remanent magnetization of the magnetic ink after magnetization by the field strength of 1.5 T at room temperature and 10 mT at 100 °C. (E) The magnetization of the magnetic ink with 50 wt% NdFeB is tested under different reprogramming magnetic strength *B_Repr_* under heating temperatures ranging from 70 to 110 °C. (F) The electric conductivity of the conductive ink with different weight fractions of carbon-based particles (carbon nanotubes: graphene = 3:1). (G) The temperature of conductive ink with 12 wt% carbon-based particles under different electric power *P_Repr_*. (H) Successive cycles reprogramming in opposite direction under *B_Repr_* = 10 mT at 100 °C.

To realize the robot’s layered structure with in situ remagnetization, magnetic actuation, and electric-resistance sensing, novel functional materials and fabrication methods need to be established and fabricated (Fig. 2A). To achieve the heat-induced solid–liquid reversible polymer, naturally existing biological molecule DL-Thioctic acid (DLT) is chosen and treated with thermal-initiated ring-opening polymerization to form a soft polymer (Fig. 2B). Ferric ions (Fe^3+^) is introduced into the polymer to work as strong complex centers with carboxylic groups to form covalent crosslinks in the polymer matrix. With the weight ratio of FeCl_3_ reaching 0.06%, a transparent and soft polymer poly(DLT-Fe) is achieved with thermodynamical stability. Then, magnetic microparticles (NdFeB) with high remnant magnetization and coercivity are uniformly dispersed into the polymer, which turns into poly(DLT-Fe)-NdFeB with temperature-responded elasticity and magnetic actuation (Fig. S1). This magnetic ink exhibits a well magnetic property with NdFeB weight fraction of 40%, 50%, and 60%, where the remanent magnetization ranges from ~5 to ~7 mT under a magnetic strength of 1.5 T (Fig. 2, C and D). By heating the ink to 100 °C and turning it into a melting state, a similar level of remanent magnetization could be achieved under a much lower magnetic field *B_Repr_* of 10 mT as it is much easier to rearrange these NdFeB particles in liquid-state ink (Fig. 2D). Higher weight fraction of NdFeB particles could enhance the remanent magnetization; however, too much NdFeB weight fraction would greatly diminish the flowability of melted polymer and reduce the remanent magnetization at low *B_Repr_*. Here, the 50 wt% NdFeB shows the best magnetizing property for heat-induced reprogramming. Stronger *B_Repr_* and higher heating temperature both enhance the remagnetization. Considering 50% as the magnetization ratio for effective actuation, heating temperature of 100 °C and *B_Repr_* of 10 mT are chosen for reprogramming (Fig. 2E). For the conductive ink, waterborne polyurethane (PU) is mixed with carbon nanotubes and graphene particles to achieve the electric conductivity, high strain-resistance response, and high flexibility [42]. Its conductivity increases with a higher mass fraction of conductive particles, while too many conductive particles leads to cracking and result in little increasing in conductivity. To balance between the conductivity and the layer’s mechanical strength, 12 wt% of carbon particles are set for the conductive ink (Fig. 2F and Fig. S2).

Based on these inks’ varied material properties, extrusion and aerosol jet 3D printing are adopted for the robot fabrication [43]. Four layers including bottom/upper PU ink, magnetic ink, and conductive ink are successively printed in a 6-claws pattern to form the layered structure, with thicknesses of 100, 620, and 50 μm, respectively (Fig. 2A, right). Nano Ag ink is printed at 2 opposite sides of the conductive layer to work as heating/sensing electrodes. The conductive layer can be rapidly heated and cooled in merely ~30 s with electricity powered on or off. To achieve the heating temperature of 100 °C, power density has been set as 0.25 W/cm^2^ for magnetic reprogramming (Fig. 2G). After 11 times of repeated heating and cooling in 600 s, the conductive layer exhibits a stable electric heating property (Fig. S3). Such layered film exhibits profound repeatable heating and reprogramming performances with a steady heating temperature and 50% remanent magnetization for over 10 times (Fig. 2H).

**Fig. 3.**
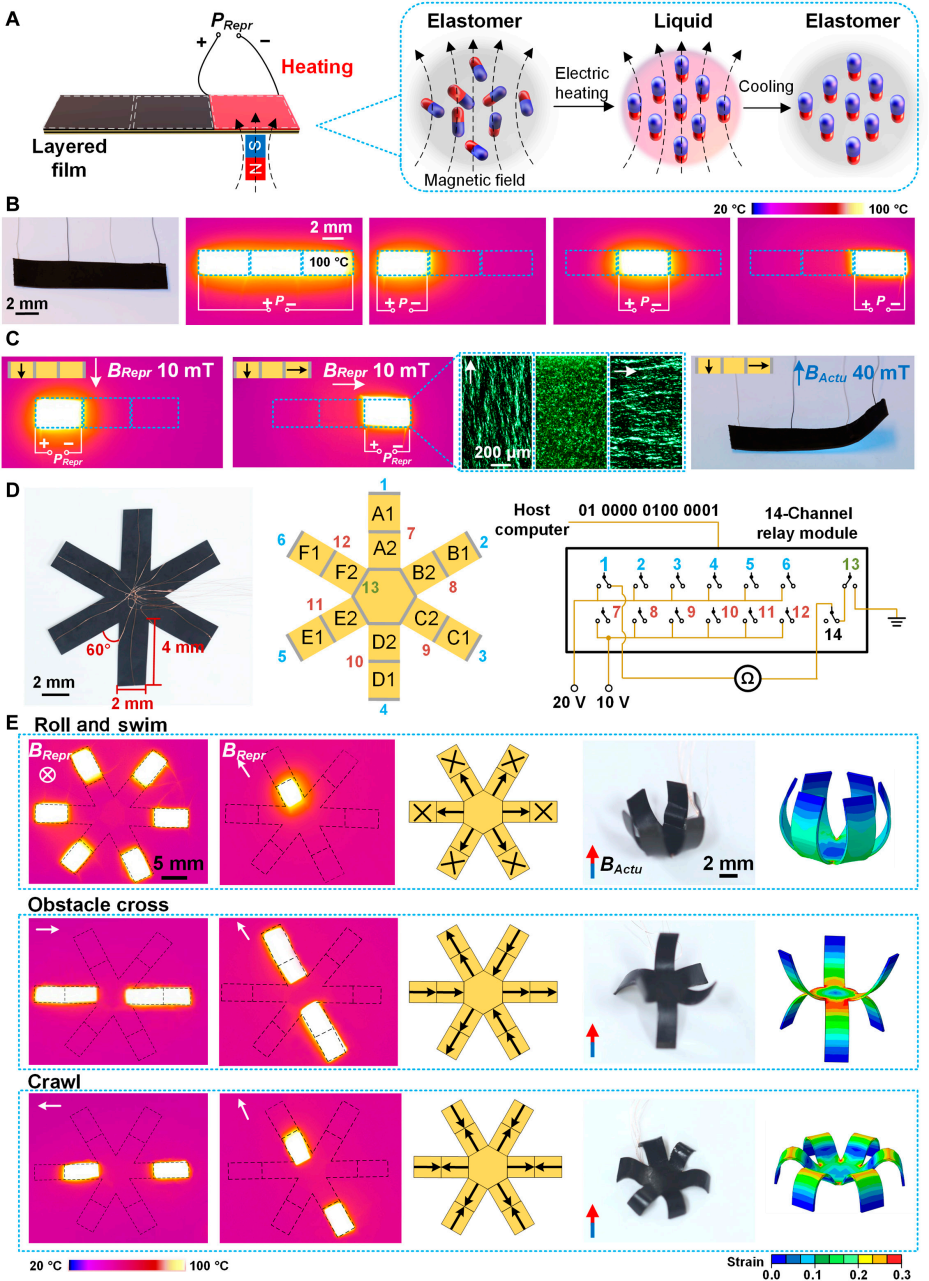
Control of heat-induced in situ reprogramming for multimode magnetic actuation. (A) The layered film of the 6-claws robot can be sectionally heated by electric power based on selected electrodes. After the magnetic layer melted, the external magnetic field *B_Repr_* is applied to realign the magnetic particles with reprogrammed remanent magnetization. (B) Layered film with 3 separated heating segments can be selectively and sectionally heated. Infrared images show that the highest temperature reaches ~100 °C. (C) By applying different direction of *B_Repr_* on the sectional heated layer, the magnetic particles exhibit distinct realignments to the *B_Repr_*, and the layered film performs the demanding deformation under an actuating magnetic field of *B_Actu_* of 40 mT. (D) The electric wire connection for robot’s 6 claws A to F, including outer electrodes No. 1 to 6, mid-electrodes No. 7 to 12, and center electrode No. 13. They are separately linked to a 14-channel relay module that can be controlled by inputting relay status signal from a host computer. Power sources (20 and 10 V) and an ohmmeter are connected to the circuit for heating and sensing. (E) Three types of reprogramming can be realized for multimode movements including roll and swim, obstacle cross, and crawl. Each section can be accurately selected for heating and reprogramming. The robot’s deformations under external actuating magnetic field *B_Actu_* agree with the finite element analysis.

To be capable of multimode deformation, the layered film is designed with a sectional pattern, where each section can be separately heated and reprogrammed into different remanent magnetization (Fig. 3A). The joints of the connected sections are printed with Ag ink to form electrodes and embedded with enameled Cu wires (80-μm diameter). By powering the selected section with electricity, it is effectively heated to ~100 °C, while the adjacent sections remain at room temperature (Fig. 3B). *B_Repr_* = 10 mT is applied on the layered film, and only the heated section could be selectively remagnetized into the required remanent magnetization (Fig. 3C, left). After repeatedly heating and applying different *B_Repr_*, the whole layered film is reprogrammed with each section exhibiting the designated remanent magnetization, which eventually achieves the in situ magnetic reprogramming and exhibits the designed deformation under 40-mT *B_Actu_* (Fig. 3C, middle and right). Under such in situ reprogramming operation, more kinds of deformations can be achieved to help the robot perform agile movements (Fig. S4).

The robot’s each claw is separated into 2 sections by outer, mid- and center electrodes to perform independent reprogramming, including outer electrodes No. 1 to 6, mid-electrodes No. 7 to 12, and No. 13 center electrode (Fig. 3D, left and middle). The electrodes are connected to a 14-channel relay module as shown in Fig. 3D (right), where each relay can be independently switched from a host computer’s signal. An ohmmeter is also connected to the module to execute the robot’s pose sensing. By logically switching these relays, the claw sections can be separately or simultaneously heated by 20- and 10-V power sources (Fig. S5). Three types of remanent magnetization patterns have been designed for the multimode motions, i.e., roll and swim, obstacle cross, and crawl. By selectively heating the claws’ sections and reprograming with *B_Repr_* (Fig. S6), the required remanent magnetization patterns are perfectly achieved for the multimode motions (Fig. 3E, left and middle). Under the actuating magnetic field *B_Actu_*, the robot performs the accurate given deformations, which agree with the corresponding finite element analysis of the programmed magnetic robot under an external magnetic field (Fig. 3E, right).

### Pose sensing of the robot with the strain-resistance effect

**Fig. 4.**
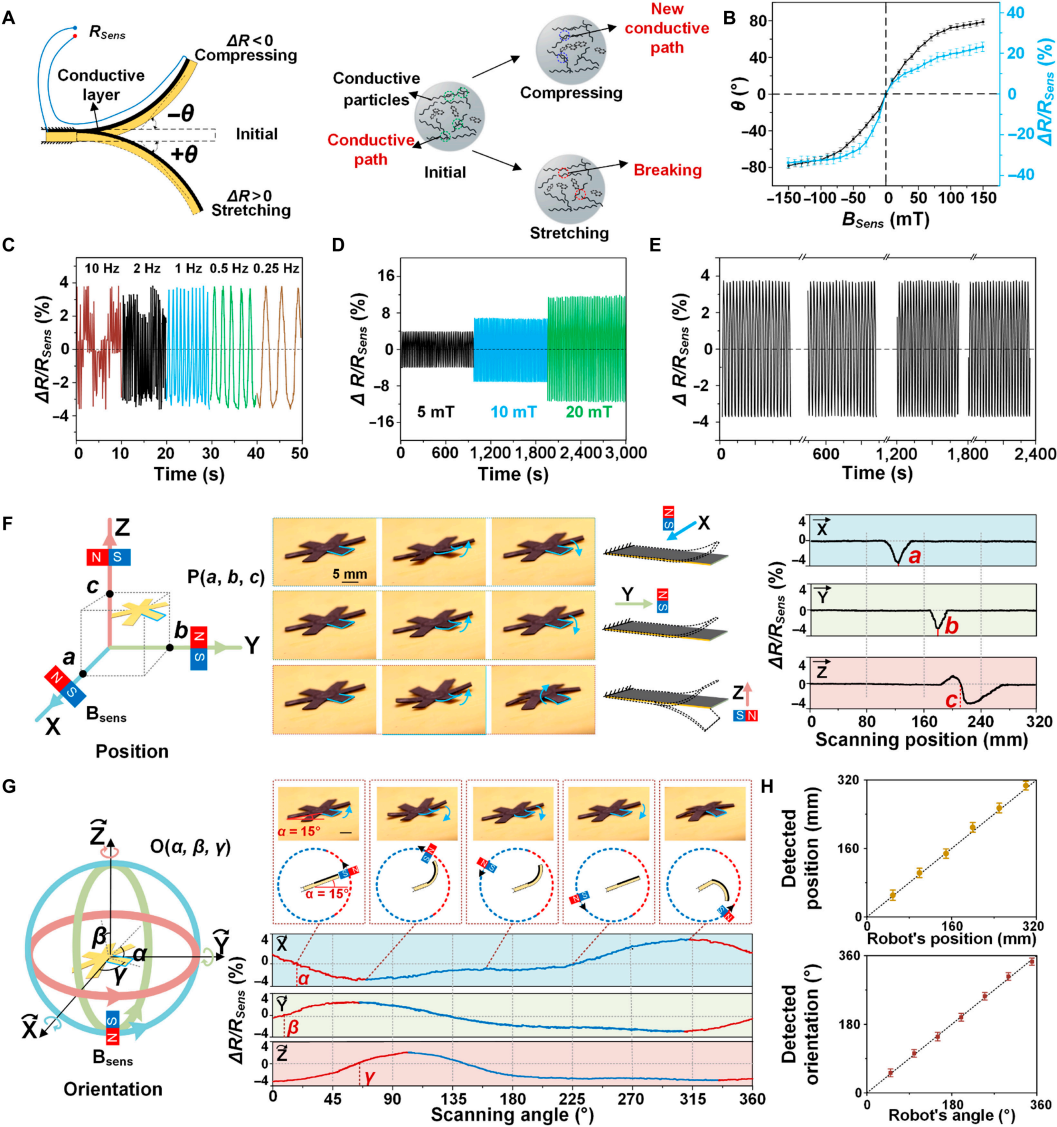
The layered film’s strain-resistance effect and pose sensing of the robot. (A) The electric resistance of the conductive layer, *R_Sens_*, varies ±*ΔR* by forming or breaking carbon particles’ conductive paths in compressing or stretching deformation, respectively. (B) The deflection of the layered film *θ* under an external sensing magnetic field *B_Sens_*, and the corresponding measured relative resistance variation *ΔR/R_Sens_*. (C) The frequency response of the layered film’s *ΔR/R_Sens_* under *B_Sens_* of 5 mT. (D and E) The stable repeatability of *ΔR/R_Sens_* after thousands of deflections under *B_Sens_* of 5, 10, and 20 mT. (F) Linearly moving *B_Sens_* in X, Y, and Z directions are separately applied on the robot to act as a scanning magnetic field in 3D space. *ΔR/R_Sens_* real-timely denotes the layered film’s deformation to locate the robot’s position in P(*a*, *b*, *c*). (G) After the robot’s position was located, the robot’s orientation O(*α*, *β*, *γ*) can be achieved by scanning rotating *B_Sens_* in X, Y, and Z directions. (H) The position and orientation of sensing *B_Sens_* and the corresponding measured robot’s pose P(*a*, *b*, *c*) and O(*α*, *β*, *γ*).

Before performing the in situ reprogramming of the robot, one critical issue is to easily acquire its posture, especially in the gastrointestinal tract with an out-of-sight environment. Luckily, the conductive layer of the robot is composed of carbon-based nanoparticles, where its conductivity is formed by the conductive paths between these nanoparticles. These conductive paths are extremely sensitive to the materials deformation as existing paths break during stretching and new paths appear in compressing, forming a strain-resistance effect on the layered film (Fig 4A, right). Since the conductive layer is printed at 1 side of the layered film, its resistance *R_Sens_* exhibits distinct change *ΔR* in compressing (*ΔR* < 0) or stretching (*ΔR* > 0) when the layered film is bent by external magnetic field *B_Sens_* (Fig. 4A, left). By applying *B_Sens_* with different strengths and directions on the robot, its claw deforms with varied bending angle *θ*, and the measured *R_Sens_* positively correlates to *θ*. Under *B_Sens_* changing from −150 to 150 mT, *θ* ranges from −79° to 79° with relative resistance variation *ΔR*/*R_Sens_* increasing from −34% to 24%, respectively (Fig. 4B). The relationship between resistance change rate *ΔR*/*R_Sens_* and bending angle *θ* can be obtained as

∆RRSens=r+Dθ−ll=Dlθ (1)

where *r* is the radius of curvature corresponding to the neutral layer and *D* is the distance from the conductive layer to the neutral layer of the layered film. According to Eq. S1, the bending angle *θ* increases with the magnetic field strength *B_Sens_*. Therefore, the resistance change rate *ΔR*/*R_Sens_* of the layered film also increases with the magnetic field strength *B_Sens_* (Eq. 1), which is in consistency with the tested results shown in Fig. 4B.

By switching on relay No. 13 and 14 in the controlling circuit (Fig. 3D, right and Fig. S5), the electric resistance between electrodes No. 1 and 13 on claw A can be rapidly and accurately measured by the ohmmeter. The response speed of measurement is limited by the mechanical property of the claw’s layered film material, which is restricted to 1 Hz (Fig. 4C). As *B_Sens_* created deformation could lead to robot movement, the deformation should be as small as possible; thus, the *B_Sens_* is set as 5 mT with maximum deformation of ±3° and *ΔR*/*R_Sens_* of ±5% (Fig. 4D). The claw of the robot exhibits a stable *ΔR*/*R_Sens_* response to repeating deformation induced by *B_Sens_* (Fig. 4E) and forms the bases of pose sensing of robot.

The pose sensing of a robot includes its position and orientation in 6 degrees of freedom. Based on the highly sensitive magnetic detection of the robot’s claw, a pose sensing strategy can be realized by linearly moving or circularly rotating an electric magnet around the robot that operates in human body (Fig. 1A). A trivial magnetic field *B_Sens_* of ~5 mT has been adopted to perform the pose detection, which is much lower than the actuating magnetic field *B_Acut_* of ~38 mT to prevent any robot movements during detecting. For its 3D position sensing, the magnet linearly scans in X, Y, and Z directions successively to create a first increasing and later decreasing magnetic strength around the robot (Fig. 4F, left). During the *B_Sens_* magnet gliding by the robot, its claws exhibit a small deformation in 1-way or 2-way bending based on different relative positions between the robot claw and magnet (Fig. 4F, middle). Therefore, *ΔR*/*R_Sens_* on claw exhibits 2 types of patterns including a “V” shape with a single spike and an “N” shape with 2 spikes (Fig. 4F, right). The “V” shape appears when the magnet linearly moves in the claw’s surface plane, here shown in X and Y directions. Its spike value indicates the largest deformation formed on claw, which happens at the moment the magnet moves to the closest distance to the robot (Movie S1). The “N” shaped *ΔR*/*R_Sens_* appears when the magnet moves perpendicular to the surface plane, here shown in the Z direction (movie S2). The *ΔR*/*R_Sens_* = 0 between the 2 spikes shows the closest distance between the robot and the magnet (Fig. S7A). Via these closest-distance conditions in both “V” and “N” *ΔR*/*R_Sens_*, the robot’s position can be located by recording the magnet’s position in the X, Y, and Z coordinates, i.e., the *a*, *b*, and *c* in Fig. 4F right, labeled as P(*a*, *b*, *c*).

For the robot’s orientation sensing, the 5-mT *B_Sens_* circularly scans in X, Y, and Z axes separately, which creates a 3D rotating magnetic field around the robot (Fig. 4G, left and Fig. S7B). During each 360° circular scanning, *ΔR*/*R_Sens_* is measured with a maximum value and a minimum value, which correspond to the claw’s largest deformation in upward and downward bending, respectively (Fig. 4G, right). The 2 spanning angles between maximum and minimum values are remarkably different due to the low strength 5-mT *B_Sens_*, as the blue and red lines shown in Fig. 4G (right). Thus, the *ΔR*/*R_Sens_* = 0 in the smaller spanning angle (red line) denotes the orientation angle of the robot, i.e., O(*α*, *β*, *γ*) (movie S3). The detected position and orientation from *B_Sens_’s* movement agree well with the robot’s actual position and orientation, with an accuracy of ± 3 mm in position and ± 2.5° in orientation (Fig. 4H). Specially, singularity situation could occur when the detecting claw is aligned with 1 scanning axis (e.g., X axis) and perpendicular to the plane (YZ plane) (Fig. S8). At this circumstance, the 2 spanning angles between Max (*ΔR/R_Sens_*) and Min (*ΔR/R_Sens_*) are identical, thus the orientation sensing strategy cannot be applied. Luckily, this octopus inspired robot with 6 claws can perform such orientation sensing on any other claw that are not align to scanning axes, and the robot’s orientation can still be achieved. Since *B_Sens_* is much lower than *B_Repr_* and *B_Actu_*, such pose sensing can effectively achieve the status of the robot with minimal impact on the robot’s remnant magnetization and motions, which is critical for the robot’s out-of-sight in situ reprogramming and is the foundation of its multimode motions.

### The complex and multimode motions of the robot based on pose sensing and in situ reprogramming

**Fig. 5.**
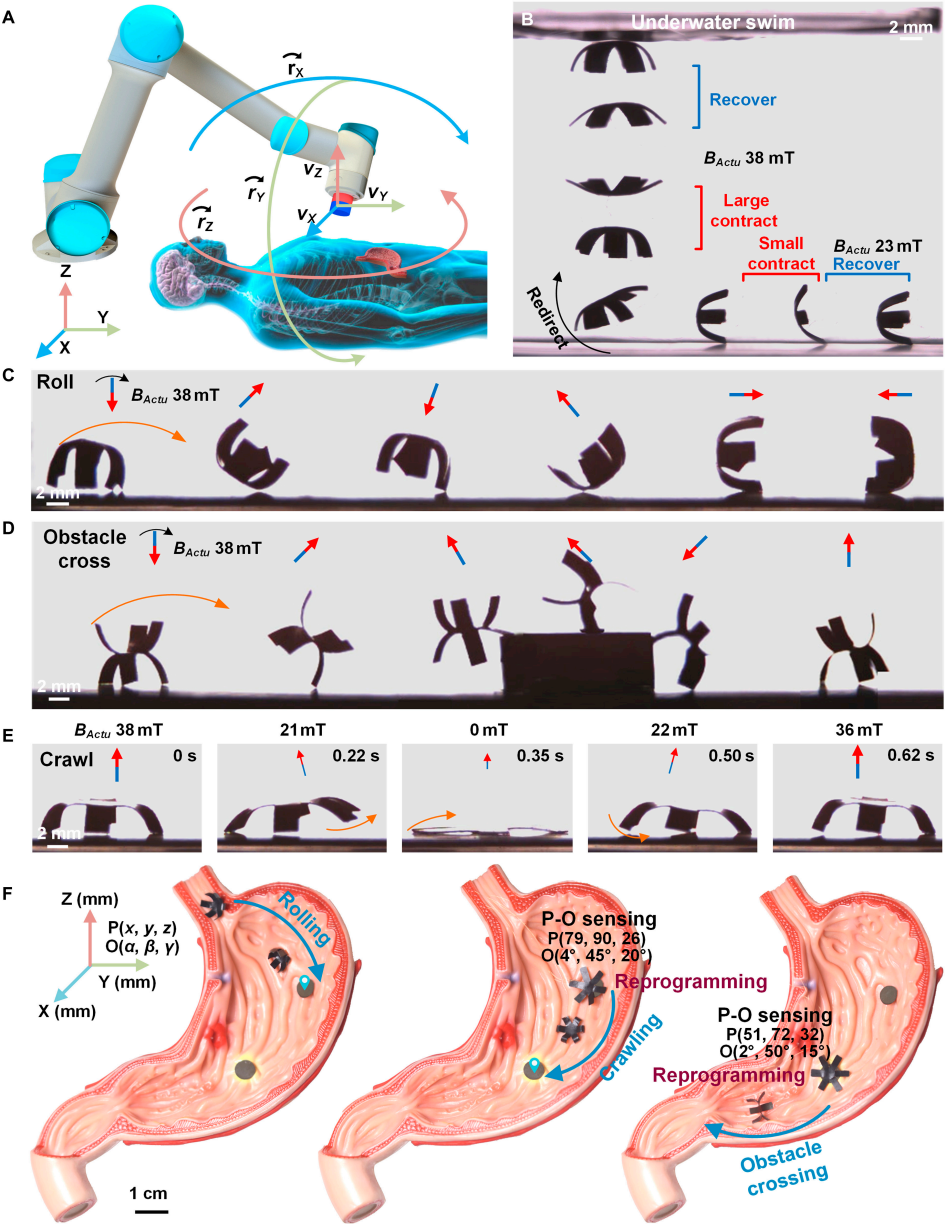
Controlling of robot’s multimode motions and its operation in the stomach model. (A) *B_Actu_*, *B_Sense_*, and *B_Repr_* could be generated by an electric magnet mounted on mechanical arms to fast and agilely move around the human body. (B to E) The multimode motions of the robot actuated by controlling the strength and direction of *B_Actu_*, including underwater swimming, rolling, obstacle crossing, and crawling. (F) The functions of pose sensing, in situ reprogramming, and multimode motions are performed in a stomach model to demonstrate the complex and accurate manipulations of robots in an out-of-sight environment.

To perform functions of in situ reprogramming, pose sensing, and multimode actuating, 3 types of external magnetic fields, *B_Repr_*, *B_Sense_*, and *B_Actu_*, are required with rapidly adjustable direction and strength. A mechanical arm with a high degree of freedom could be mounted with an electric magnet to be capable of generating such large reachable, accurate, and rapid positioning magnetic fields (Fig. 5A). After reprogramming the robot into different patterns of remnant magnetization, varied types of motions have been achieved by modulating the *B_Actu_* (Fig. S9). For the roll and swim pattern, a pulsating *B_Actu_* could shift its deformation between contracting and thin-film states, where it can freely swim underwater by adjusting the strength and direction of pulsating *B_Actu_* (Fig. 5B and Movie S4). With a constant but rotating *B_Actu_*, such a magnetization pattern maintains a contracting state to easily and quickly roll on a surface (Fig. 5C and movie S5). By reprogramming the robot’s adjacent arms into opposite magnetization, an obstacle cross movement can be created under a rotating *B_Actu_*, where the outward-spreading arms help it expand its space to be more capable of rolling over high barriers (Fig. 5D and movie S6). After magnetizing the outer and inner sections in opposite directions, a crab-like crawling movement is realized under a swing and pulsating *B_Actu_* with high loading capacity (Fig. 5E and Movie S7). These multimode motions created by in situ reprogramming provide the robot with high adaptivity for complex and multifunctional operations. Combining the robot’s pose sensing, a more controllable and accurate reprogramming and operating can be achieved in the out-of-sight gastrointestinal environment, which could help realize more complex functions of drug delivery and release, tissue biopsy (Fig. 5F and Movie S8).

## Discussion

To meet the robot’s urgent demands of in situ reprogramming, multimode motions and the necessary pose sensing, a 6-claws magnetic-driven robot is designed and fabricated in a multilayer structure with a magnetic-driven layer covered by a heating-sensing conductive layer. The heating-sensing layer can selectively and segmentally heat the magnetic-driven layer into solid–liquid transition for a much lower remagnetization field strength of *B_Repr_* ~10 mT, to achieve the in situ magnetization reprogramming and multimode motions. Such heating-sensing layer also possesses an electrical-resistance effect and can detect the robot’s position and orientation by creating small deformation of its claw under sensing field *B_Sens_* with a low strength of ~5 mT. Under the integration of in situ reprogramming and pose sensing, an out-of-sight accurate manipulation is realized on the robot with multimode motions, including swimming, rolling, crawling, and obstacle crossing. The strategies and theories have been developed for in situ reprogramming and pose sensing, and the applications in gastroscopy have been demonstrated. This magnetic-driven robot paves the way for the development of more precise and sophisticated gastrointestinal medical manipulations.

## Materials and Methods

### Preparation of printing inks

Magnet ink was prepared with 10 g of DLT (Macklin, D819096) heated in an oil bath to 70 °C to get a liquid-like melt. FeCl_3_ (Macklin, I811935) with a mass ratio 0.06% of DLT was added into the melt and magnetically stirred for 5 min to get a transparent and soft polymer poly(DLT-Fe) as the host gel. Then, it was heated to 90°C and added with varied mass ratio NdFeB microparticles (Magnequench, MQFP-B-20052-089) under stirring for 50 min to ensure homogeneous dispersion. The melt mix can be cooled below 70 °C to be capable of extrusion printing. Conductive ink was prepared with carbon nanoparticles mixed in the PU (Macklin A909856) at 12%wt and ultrasonic for 2 hours for aerojet printing. The carbon nanoparticles include carbon nanotubes and graphene with a mass ratio of 3:1.

### Fabrication of the 6-claws robot with multilayer structure

The desktop robot (JR3300, JANOME) was used for the 3D printing. PU ink (5 ml) was loaded into the pneumatic atomizer of the aerosol jet printer and printed with a 410-μm nozzle. The flow rates of carrier and sheath gas were set to 1,700 and 300 ccm, and the printing speed and number of printing layers were set to 6 mm/s and 3 passes. Then, the PU layer was printed and sintered at 50 °C for 5 min. The magnet ink was put into a heatable 30 cc syringe barrels and printed with a 620-μm diameter nozzle, printing temperature of 70 °C and printing pressure of 0.4 Mpa. Another PU layer was printed on the magnetic-driven layer for sealing. Ag ink (BroadCON-INK550, BroadTeko) was printed on the PU layer with a 150-μm-diameter nozzle to work as electrodes (Fig. 3D). The flow rates of carrier and sheath gas were set to 1,500 and 400 ccm. Cu wires with diameter of 80 μm were connected to Ag electrodes for heating power and electrical resistance sensing. The conductive ink was printed over using a 410-μm nozzle with flow rates of carrier 2,000 ccm and sheath gas 300 ccm. The print speed and print passes were set to 6 mm/s and 3 passes.

### Characterization and measurements

The microstructures of the layered film were inspected by scanning electron microscopy (SEM) (TESCAN) operated at 5 kV. The magnetic hysteresis loop of the layered film was measured using a vibrating sample magnetometer (Lake Shore 7410) with external magnetic field sweeping from −2 to +2 T. The conductivity and heating properties of the heating-sensing conductive layer were measured with a Four-Point Probe and a thermal infrared imager, respectively. The resistance of the layered film was measured by a source meter (2400, Keithley) with voltage powered by a dc power (HSPY-600, Beijing Hansheng Puyuan Instrument Co. Ltd.). The external magnetic field was generated by a 3-axis Helmholtz coil (Fig. S10). Magnetic fields with different strength and direction are controlled by a signal generator and amplifier (Fig. S7).

### Control of heat-induced in situ reprogramming of robot

For the 6-claws robot, each part needs to be separately magnetized for its multimode motion under external magnetic field. The electrodes were connected to a 14-channel relay array module, where each segment of conductive layer can be selectively heated by 20- or 10-V electrical power by inputting relay status from a hosting computer (Fig. S5). For example, to achieve the swimming and rolling motion (Fig. 3E, upper panel and Fig. S6A), segments A1, B1, C1, D1, E1, and F1 need to be firstly heated, and 0x1FFF (0b01 1111 1111 1111) are input to the relay module to connect electrodes 1 to 6 to 20 V and electrodes 7 to 12 to 10 V to create electricity between them and heat these segments. Then, the corresponding magnetic-driven layer turns into melting state, and a vertical downward magnetic field *B_Repr_* 10 mT is applied on the robot to remagnetize these segments. Similar processes are repeatedly applied on A2 to F2 segments by controlling the relay module for heating and changing the direction of *B_Repr_* (Fig. S6A). Then, the in situ reprogramming of robot into swimming and rolling mode is realized. With such method, the obstacle crossing and crawling modes can be achieved as shown in Fig. 3E and S6.

### Finite element analysis

In the finite element analysis, the robot deformation in response to the actuation magnetic fields were simulated by a user-defined element subroutine. It was proposed by Kim et al. [18] and modified in this study, implemented in the commercial finite element analysis software ABAQUS. The residual magnetization of the robot was set as 245 kA/m, and the layered film was set as linear elastic with Young’s modulus of 1 MPa and Poisson’s ratio of 0.49. The density was measured and set as 4.2 g/cm^3^, and the actuation uniform magnetic field is 150 mT.

### Control of pose sensing of robot

The robot's pose detection experiment was conducted in a transparent box in size of 320 * 320 * 320 mm. A magnet was placed on the guide rail and moved in parallel in the X, Y, and Z directions with a speed of 40 mm/s, and a total of 24 s has been scanned in 3 directions. The change of electric resistance of the robot was measured in real time with a ohmmeter. The robot’s orientation detection was performed in a Helmholtz coil, which generates a rotated magnet field around the robot with frequency of 0.05 Hz, a total of 60 s have been completed in all 3 directions.

## Data Availability

All data needed to evaluate the conclusions in the paper are present in the paper and/or the Supplementary Materials.

## Supplementary Materials

Supplementary 1 Fig. S1. SEM images of the magnetic microparticles and elemental mapping of the magnet material. Fig. S2. SEM characterization of the conductive materials. Fig. S3. Heating stability test of the conductive layer upon the repeated applied power density of 0.25 W/cm^2^. Fig. S4. Various kinds of deformations created by in situ reprogramming. Fig. S5. The control of heating-induced in situ magnetization reprogramming. Fig. S6. Distributed heating and reprogramming of 6-claws robot. Fig. S7. Detecting strategy of position sensing and orientation sensing. Fig. S8. During the measurement process, singularity may occur when encountering special angles. Fig. S9. Actuation magnetic fields of different motion modes. Fig. S10. The customized system controls the magnetic robot for in situ reprogramming and multimode motions.

Supplementary 2 Movie S1. Experimental results of X-axis position detection (MP4). Movie S2. Experimental results of Z-axis position detection (MP4). Movie S3. Experimental results of Y-axis orientation detection (MP4). Movie S4. The 6-claws robot's horizontal movement and vertical swimming underwater (MP4). Movie S5. The 6-claws robot rolling on a plane under the action of a rotating magnetic field (MP4). Movie S6. The 6-claws robot crawling on plane surface (MP4). Movie S7. The 6-claws robot climbing over obstacles (MP4). Movie S8. Multimode motion of the 6-claws robot in the stomach model (MP4).
