# Supplementary material for: A Magnetic-Driven Multi-motion Robot with Position/Orientation Sensing Capability
Source: Research (Wash D C). 2023 Jun 21;6:0177. doi: 10.34133/research.0177 (PMC11778601; doi:10.34133/research.0177)
Supplement: Supplementary 1 — Fig. S1. SEM images of the magnetic microparticles and elemental mapping of the magnet material. Fig. S2. SEM characterization of the conductive materials. Fig. S3. Heating stability test of the conductive layer upon the repeated applied power density of 0.25 W/cm2. Fig. S4. Various kinds of deformations created by in situ reprogramming. Fig. S5. The control of heating-induced in situ magnetization reprogramming. Fig. S6. Distributed heating and reprogramming of 6-claws robot. Fig. S7. Detecting strategy of position sensing and orientation sensing. Fig. S8. During the measurement process, singularity may occur when encountering special angles. Fig. S9. Actuation magnetic fields of different motion modes. Fig. S10. The customized system controls the magnetic robot for in situ reprogramming and multimode motions. [file research.0177.f1.docx]

Supporting Information

**A Magnetic-Driven Multi-Motion Robot with Position/Orientation Sensing Capability**

Liwen Zhang, Song Zhao, Xinzhao Zhou, Xueshan Jing, Yu Zhou, Yan Wang, Yantong Zhu, Xiaolin Liu, Zehui Zhao, Deyuan Zhang, Lin Feng, Huawei Chen*

School of Mechanical Engineering and Automation, Beijing Advanced Innovation Center for Biomedical Engineering, Beihang University, Beijing 100191, China

*^*^Corresponding Authors: Tel: +86-10-82339717*

E-mail: chenhw75@buaa.edu.cn (H.W.Chen)

**Supplemental Information**

**The six-claws robot’s deformation induced by *B_Sens_* and its relation with the change of electric resistance**

Since the conductive layer is printed at one side of the layered film, it’s resistance *R_Sens_* exhibits distinct change *ΔR* in compressing (*ΔR* < 0) or stretching (*ΔR* > 0) when the layered film is bent by external magnetic field *B_Sens_*. By applying *B_Sens_* with different strengths and directions on the robot, its claw deforms with varied bending angle *θ*, and the measured *R_Sens_* positively correlates to ***θ***. Therefore, the relationship between *θ*, magnetic field strength *B_Sens_* and resistance change rate ***ε*** is established.

An effective magnetic moment *M_net_* was produced due to the interaction of *B_Sens_* and patterned magnetization profile *m*, resulting in a rotation of the layered film. The layered film transformation is actively driven by magnetic torque (*T_m_*) and passively resisted by deformation-induced elastic torque (*T_e_*). Since the magnetic torque and elastic torque reach a balance under the actuating field *B_Sens_*, a target shape of the layered film forms. The layered film along the neutral axis (*x* axial) can mesh into infinitesimal elements. The total rotation angle *θ* at position *l* be expressed as

$$\begin{aligned} \theta=\int_{0}^{l} \int_{0}^{s} \frac{m\times B_{Sens}A}{EI}dsds\#\text{S1} \end{aligned}$$

in which, *A*, *E*, and *I* represent the cross-sectional area of an arbitrary element, Young’s modulus, and the area moment of inertia, respectively.[1-3]

For the conductive layer, the resistance changes when the bending deformation occurs, and the resistance change rate can be expressed as

$$\begin{aligned} \frac{\Delta R}{R_{Sens}}=\frac{\rho\frac{\Delta l}{S}}{\rho\frac{l}{S}}=\frac{\Delta l}{l} \end{aligned}$$

where *ρ* is the resistivity, *l* the length of the material, and *S* the cross-sectional area of the material. The curvature *k(s)* at position *s* can be calculated as

$$\begin{aligned} k\left( s \right)=\frac{d\theta\left( s \right)}{ds}=\frac{M_{net}\times B_{Sens}}{EI} \end{aligned}$$

The radius of curvature corresponding to the neutral layer can be calculated as

$$r=\frac{1}{k}$$

Finally, the relationship between resistance change rate *ΔR*/*R_Sens_* and bending angle *θ* can be obtained as

$$\begin{aligned} \frac{\Delta R}{R_{Sens}}=\frac{(r+D)\theta-l}{l}=\frac{D}{l}\theta\#\text{S2}\# \end{aligned}$$

where *D* is the distance from the conductive layer to the neutral layer of the layered film.

According to equation S1 and S2, when *m* of layered film is given, the bending angle *θ* increases with the magnetic field strength *B_Sens_*. Therefore, the resistance change rate *ΔR*/*R_Sens_* of the layered film also increases with the magnetic field strength *B_Sens_*, which is well consistent with the experimental results shown in Figure 4B.


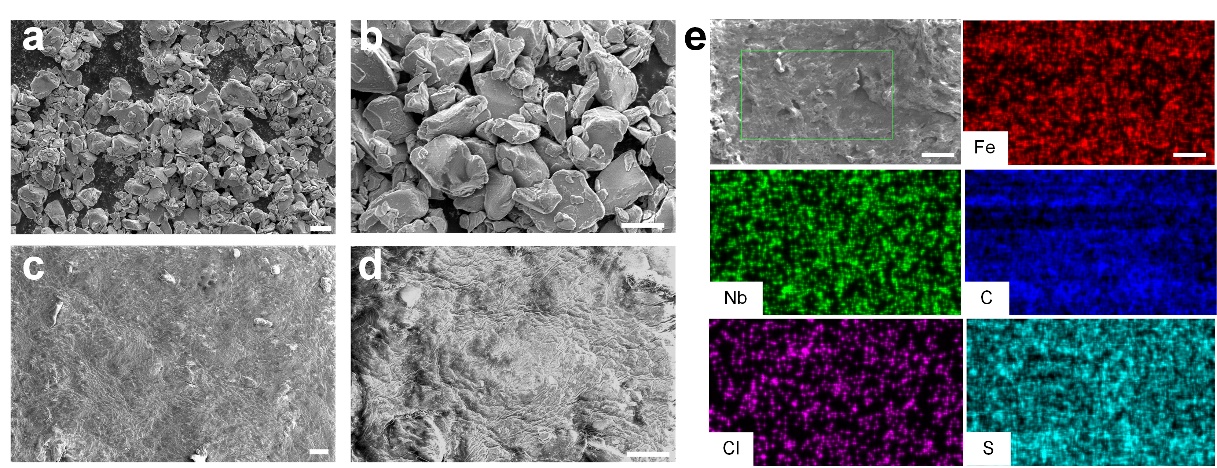


**Figure S1.** SEM images of the magnetic microparticles and elemental mapping of the magnet material. (a-b) The SEM images of the used magnetic microparticles (average size of 5 μm). Scale bars, 20 μm. (c-d) Cross-sectional morphology of the magnetic-driven layer with scale bar of 100 μm c, and 50 μm. (e) SEM characterization and elemental mapping of the HME material. The elemental mapping of Fe, Nb, C, Cl, and S elements on a randomly selected area of the HME cross-section. Scale Bars: 200 nm. The SEM images and the elemental mapping results both confirmed the uniform dispersion of NdFeB microparticles.


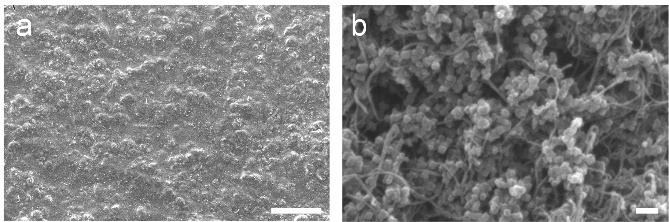


**Figure S2.** SEM characterization of the conductive materials. (a) Morphology of the conductive layer with scale bar of 100 μm. (b) The SEM images of the carbon-based conductive particles. Scale bars, 200 nm.


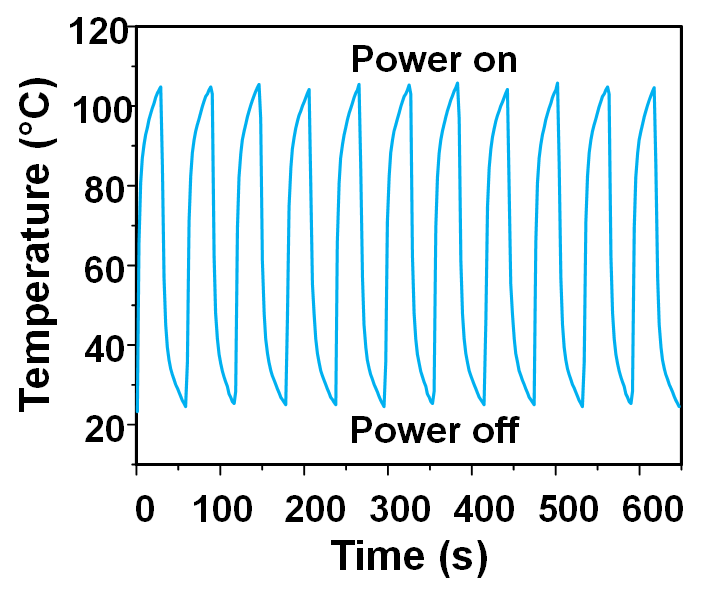


**Figure S3.** Heating stability test of the conductive layer upon the repeated applied power density of 0.25 W/cm^2^.


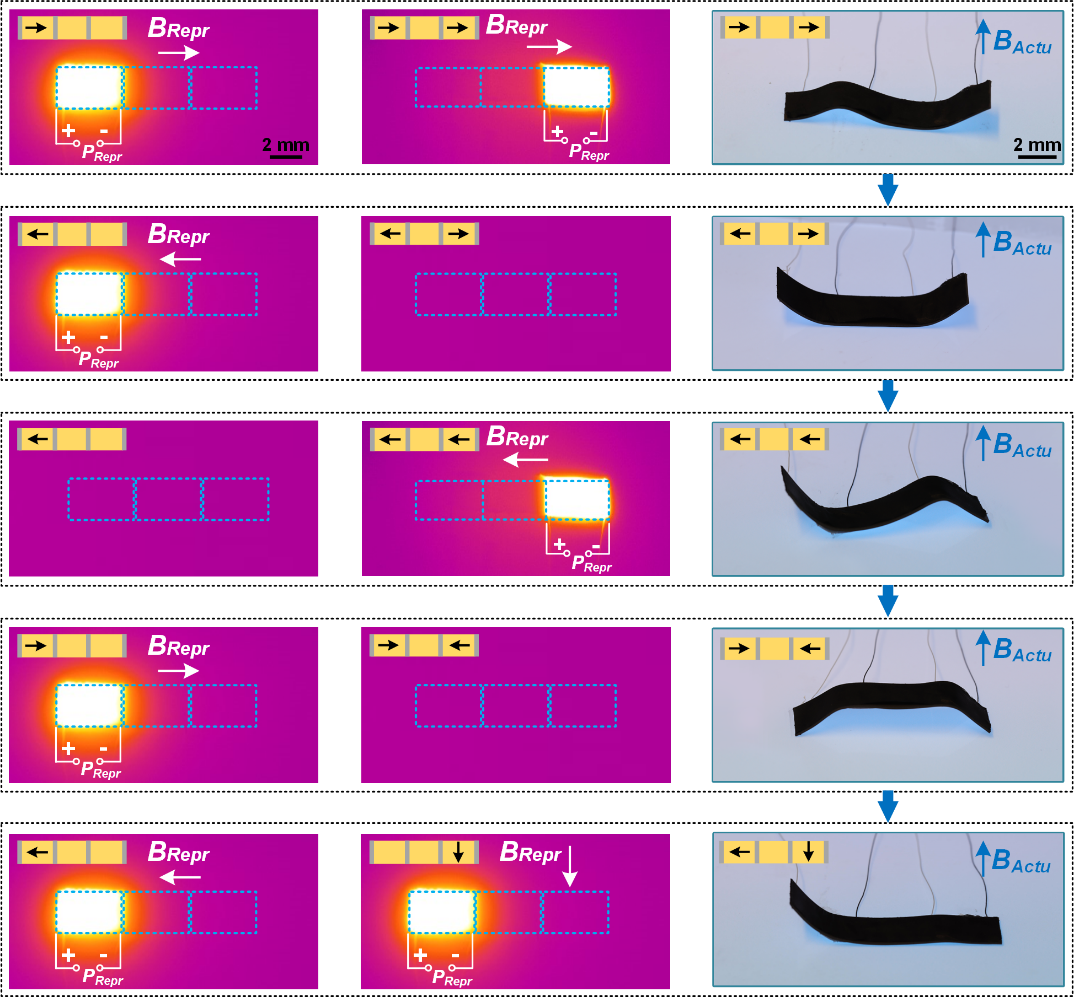


**Figure S4.** Various kinds of deformations created by *in situ* reprogramming. The layered film could be *in situ* reprogrammed with remanent magnetization in different directions to achieve varied actuating deformations under the same *B_Actu_*.


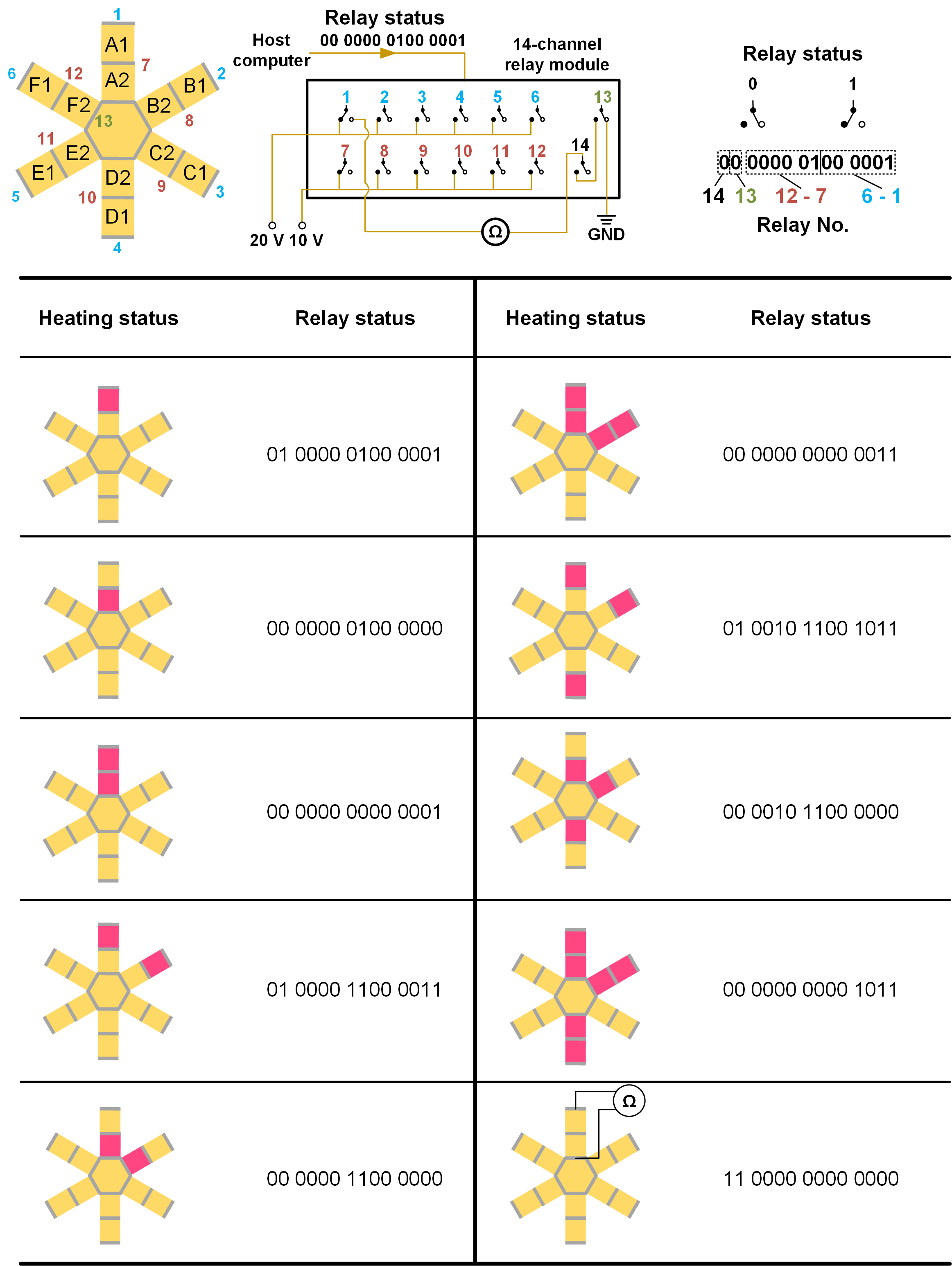


**Figure S5.** The control of heating-induced *in situ* magnetization reprogramming. 13 electrodes on the six-claws robot are connected to a 14-channel relay module, which can receive a host computer signal to distribute electrical heating power over the robot. With different segments heated, varied magnetization pattern can be achieved on the robot.


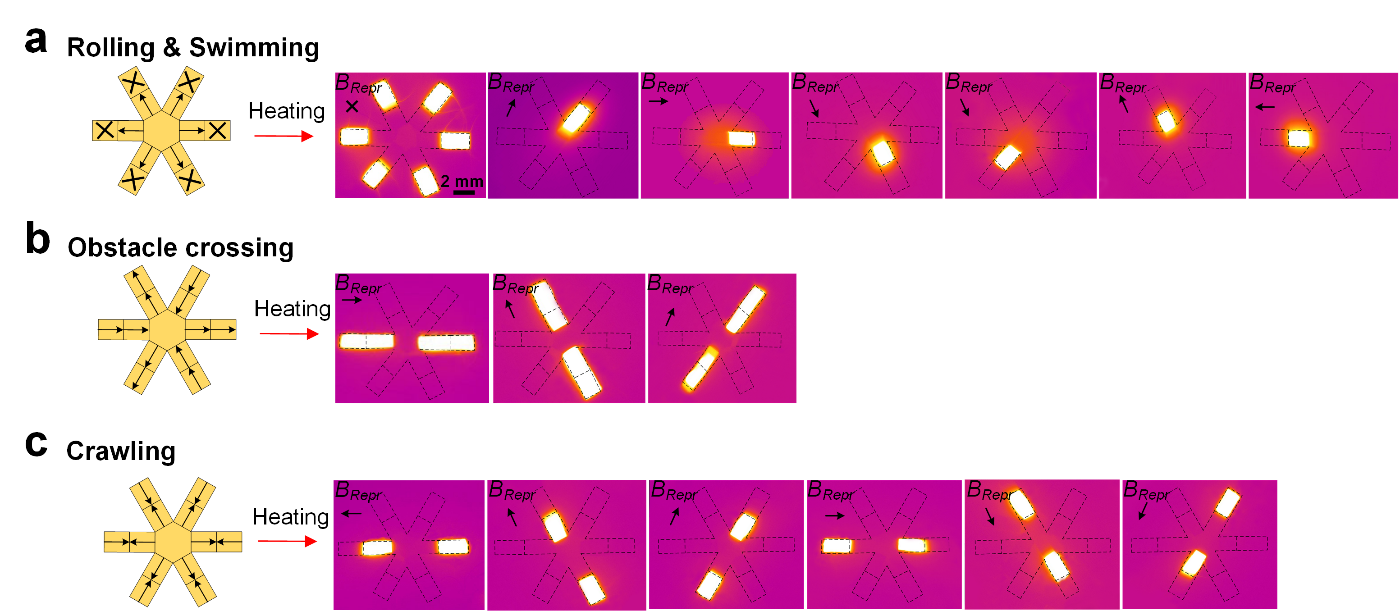


**Figure S6.** Distributed heating and reprogramming of six-claws robot. (a) For rolling & swimming state, first heat the outermost part of the grab and apply a uniform magnetic field. Then distribute heating and apply corresponding magnetic field to each part. (b) Obstacle crossing state. (c) Crawling state.


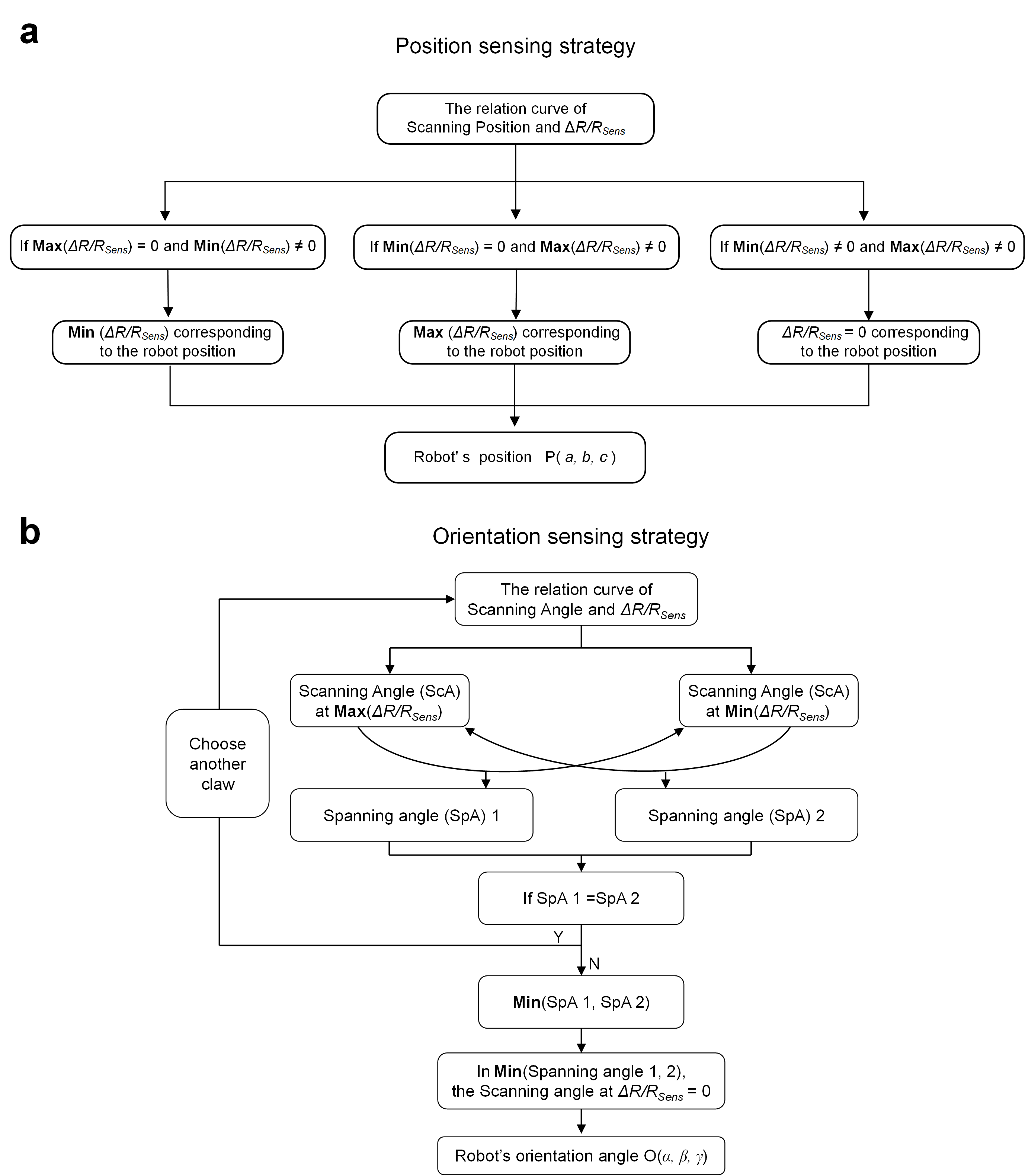


**Figure S7.** Detecting strategy of position sensing and orientation sensing. (a) Position sensing strategy. *ΔR*/*R_Sens_* on claw exhibits two types of patterns, e.g. a “V” shape with a single spike and an “N” shape with two spikes (Fig. 4F). In “V” shape, a **Max**(*ΔR/R_Sens_*) or **Min**(*ΔR/R_Sens_*) appears at spike and their corresponding scanning position can be treated as robot position. In “N” shape, **Max**(*ΔR/R_Sens_*) and **Min**(*ΔR/R_Sens_*) appear at the two spikes, and the *ΔR/R_Sens_* = 0 between **Max**(*ΔR/R_Sens_*) and **Min**(*ΔR/R_Sens_*) represents the robot position. Via this position sensing strategy, the robot’s position can be located by recording the magnet’s position in the X, Y and Z coordinates, i.e. the *a*, *b* and *c* in labeled as P(*a*, *b*, *c*). (b) Orientation sensing strategy. *ΔR*/*R_Sens_* is measured with a maximum and minimum values, i.e. **Max**(*ΔR/R_Sens_*) and **Min**(*ΔR/R_Sens_*), which correspond to the claw’s largest deformation in upward and downward bending, respectively (Fig. 4G). The two spanning angles between **Max**(*ΔR/R_Sens_*) and **Min**(*ΔR/R_Sens_*) are usually different due to the low strength *B_Sens_*, named as spanning angle (SpA) 1 and SpA 2. The Δ*R*/*R_Sens_* = 0 in the smaller spanning angle **Min**(SpA 1, SpA 2) denotes the orientation angle of the robot, thus the robot orientation O(*α*, *β*, *γ*) can be achieved. Specially, at singular position with SpA 1 = SpA 2, selecting another adjacent claw could detect the robot’s orientation.


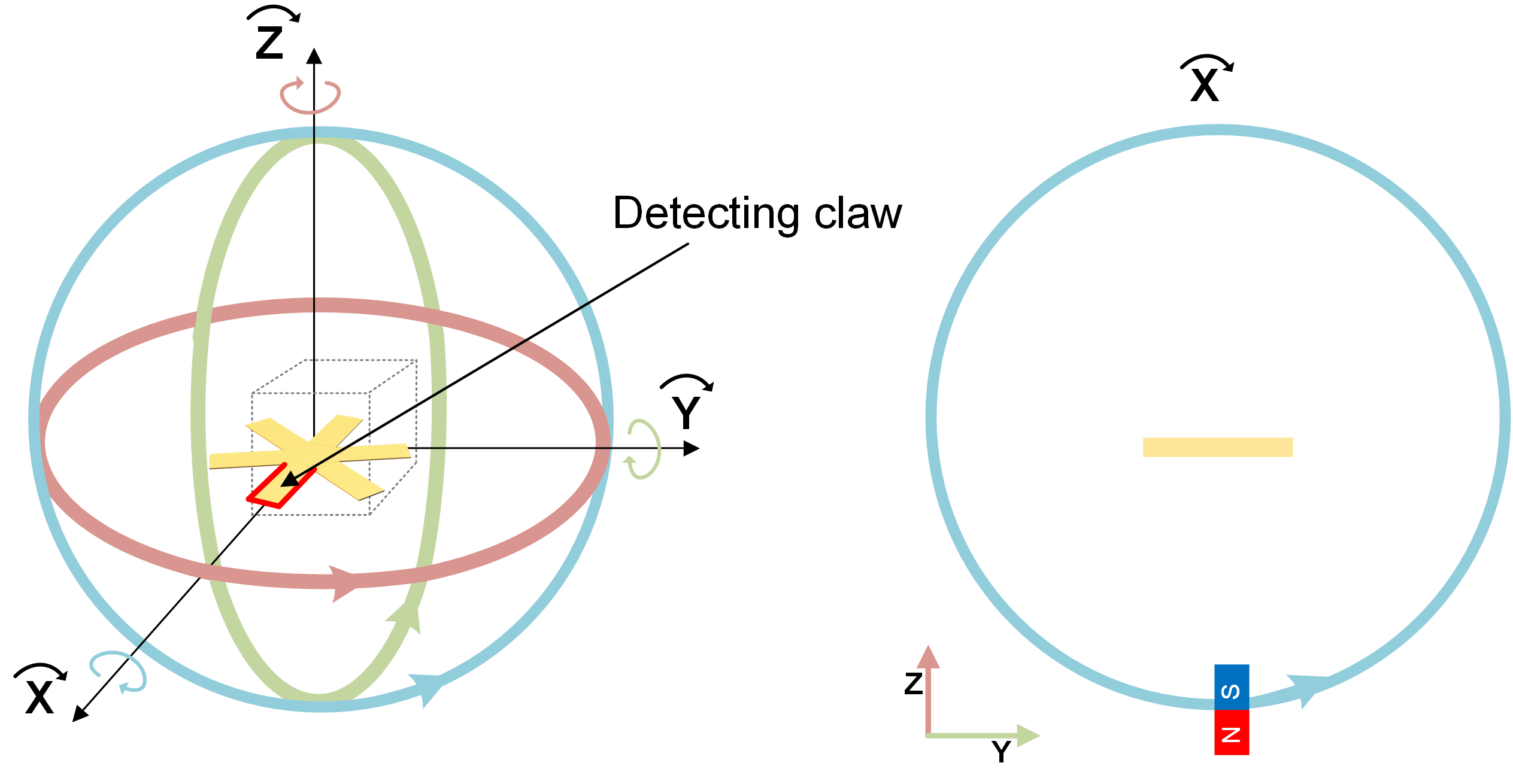


**Figure S8.** Singularity situation could occur when the detecting claw is aligned with one scanning axis (e.g. X axis) and perpendicular to the plane (YZ plane) (Fig. S8). At this circumstance, the two spanning angles between Max(*ΔR/R_Sens_*) and Min(*ΔR/R_Sens_*) are identical, thus the orientation sensing strategy cannot be applied.


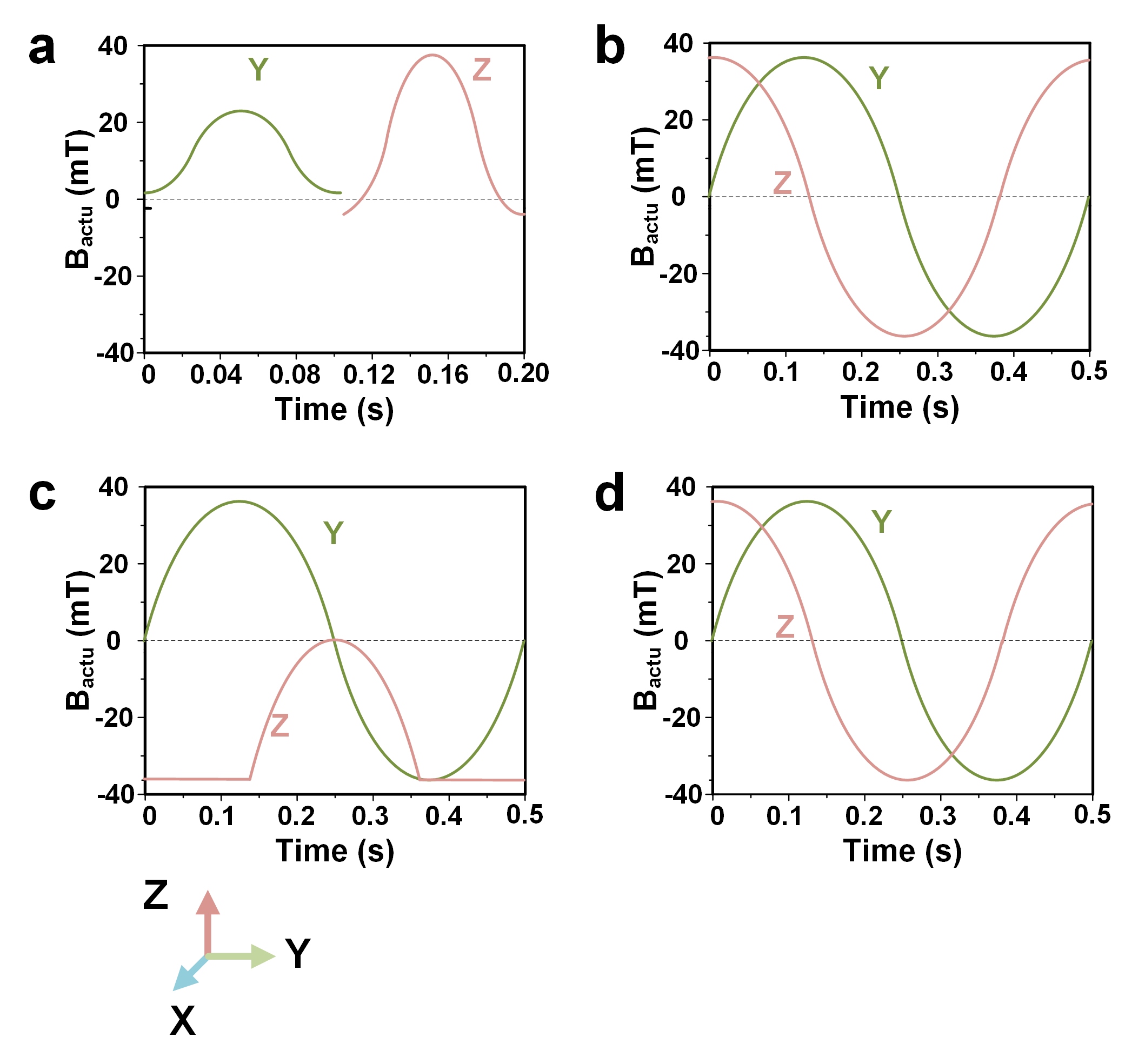


**Figure S9.** Actuation magnetic fields of different motion modes. (a) For the swimming state, the corresponding magnetic field is applied in the Y-axis direction, and the small phase transition moves in the horizontal direction. Apply the corresponding magnetic field in the Z-axis direction, and the large phase transition moves in the vertical direction. (b) For the rolling state, the two sinusoidal magnetic fields of Y axis and Z axis are combined to form the rotating magnetic field on the YZ plane. (c) For crawling state, Y-axis and Z-axis magnetic fields are compounded. (d) For obstacle crossing state, apply the rotating magnetic field of YZ plane.


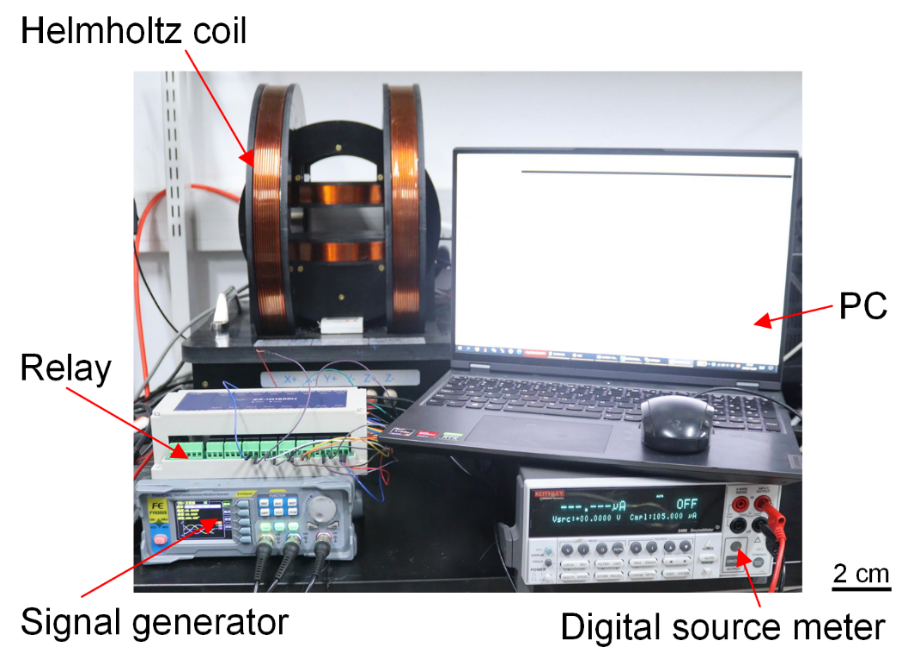


**Figure S10.** The customized system controls the magnetic robot for *in situ* reprogramming and multimode motions.

**References**

1. Chen, Z., Lin, Y., Zheng, G., Yang, Y., Zhang, Y., Zheng, S., Li, J., Li, J., Ren, L., and Jiang, L. (2020). Programmable Transformation and Controllable Locomotion of Magnetoactive Soft Materials with 3D-Patterned Magnetization. ACS Appl. Mater. Interfaces 12, 58179-58190.

2. Kim, Y., Parada, G.A., Liu, S., and Zhao, X. (2019). Ferromagnetic soft continuum robots. Sci. Robot. 4, eaax732.

3. Zhang, Y., Wang, Q., Yi, S., Lin, Z., Wang, C., Chen, Z., and Jiang, L. (2021). 4D Printing of Magnetoactive Soft Materials for On-Demand Magnetic Actuation Transformation. ACS Appl. Mater. Interfaces 13, 4174-4184.
